# Sex-Dependent Signaling Pathways Underlying Seizure Susceptibility and the Role of Chloride Cotransporters

**DOI:** 10.3390/cells8050448

**Published:** 2019-05-13

**Authors:** Pavel A. Kipnis, Brennan J. Sullivan, Shilpa D. Kadam

**Affiliations:** 1 Neuroscience Laboratory, Hugo Moser Research Institute at Kennedy Krieger, Baltimore, MD 21205, USA; Kipnis@kennedykrieger.org (P.A.K.); SullivanB@kennedykrieger.org (B.J.S.); 2 Department of Neurology, Johns Hopkins University School of Medicine, Baltimore, MD 21205, USA

**Keywords:** seizures, epilepsy, refractory, chloride, neurotrophins, neurosteroids, neonatal, developmental disorders.

## Abstract

Seizure incidence, severity, and antiseizure medication (ASM) efficacy varies between males and females. Differences in sex-dependent signaling pathways that determine network excitability may be responsible. The identification and validation of sex-dependent molecular mechanisms that influence seizure susceptibility is an emerging focus of neuroscience research. The electroneutral cation-chloride cotransporters (CCCs) of the *SLC12A* gene family utilize Na^+^-K^+^-ATPase generated electrochemical gradients to transport chloride into or out of neurons. CCCs regulate neuronal chloride gradients, cell volume, and have a strong influence over the electrical response to the inhibitory neurotransmitter GABA. Acquired or genetic causes of CCCs dysfunction have been linked to seizures during early postnatal development, epileptogenesis, and refractoriness to ASMs. A growing number of studies suggest that the developmental expression of CCCs, such as KCC2, is sex-dependent. This review will summarize the reports of sexual dimorphism in epileptology while focusing on the role of chloride cotransporters and their associated modulators that can influence seizure susceptibility.

## 1. Introduction

During development, the polarity of GABAergic neurotransmission undergoes a switch from depolarizing to hyperpolarizing [1,2]. This developmental switch occurs concurrently with an increase in expression of the chloride extruder KCC2, and a decrease in [Cl^-^]_i_. The dynamic regulation of chloride is regulated primarily through the actions of NKCC1 and KCC2. The maintenance of low intracellular chloride levels is critical for GABAergic inhibition and prevents hyperexcitability, such as seizures. Fast excitatory neurotransmission is controlled by a number of factors, including AMPA receptors (AMPARs) [3]. AMPARs are heteromultimeric transmembrane proteins which are composed of the glutamate receptor subunits GluA1-GluA4. The inclusion of GluA2 into the heteromer results in Ca^2+^ impermeability through the channel. Developmentally, changes in the subunit composition of AMPARs on neonatal neurons have been observed. Neonatal neurons lack the GluA2 subunit until the third postnatal week in rodents making them Ca^2+^ permeable and is one of the underlying mechanisms that permits increased excitability and susceptibility to seizures in neonates [4]. In adults, most AMPARs are not Ca^2+^ permeable as they contain a GluA2 subunit. During postnatal development, the risk for having seizures is higher than in other periods in life [5]. Neonatal seizures are the most frequent clinical manifestation of central nervous system dysfunction in the newborn, with an incidence of 1.5–3.5/1000 in term newborns, and an incidence as high as 130/1000 in preterm newborns [5,6,7]. 

Current data from animal and human studies suggest that neonatal seizures themselves may worsen brain injury, decrease the threshold for subsequent seizures, and result in poor long-term neurologic outcomes [2,6,8,9]. Human studies have demonstrated that neonates that experience seizures have an increased risk for developing epilepsy later in life, such as temporal lobe epilepsy (TLE), the most common form of adult epilepsy with chronic recurrent spontaneous seizures originating from the temporal lobe. Both neonatal hypoxic-ischemic encephalopathy (HIE) and TLE related seizures are known to be refractory to antiseizure medicines (ASM) [10]. It is recognized that potassium chloride cotransporter 2 (KCC2), the chief neuronal Cl^-^ extruder, is downregulated in patients with refractory TLE [11,12]. The downregulation of KCC2 increases neuronal excitability and promotes epileptogenesis [13,14,15]. Additionally, overexpression of sodium potassium chloride cotransporter 1 (NKCC1), the chief neuronal Cl^-^ importer, is implicated in phenobarbital (PB) unresponsive seizures in children with autism [16,17]. It is suspected that KCC2 hypofunction and/or NKCC1 hyperfunction may be a critical mechanism behind refractory seizures due to its disruption of GABAergic networks [12]. In preclinical studies, flupirtine, a KCNQ potassium channel opener, was efficacious in reducing seizure burden in neonatal rat pups with hypoxic-ischemic insults [18] indicating a role of KCNQ in neonatal seizures. Levetiracetam has been shown to decrease seizures in adult rats with a history of neonatal hypoxia [19]. However, neither study considered sex as a biological variable [18,19].

Although a variety of models have been used to study human brain development, the most widely studied and validated models are rodents between P7 and P14, as these ages are believed to correspond to full-term babies and adolescents respectively (for further details, please see Nardou et al., 2013) [2]. Accurately identifying the etiology and developing evidence-based treatments for epilepsy remains critical. HIE is the most common origin of neonatal seizures [20]. ~50% of HIE-associated seizures are pharmacoresistant to ASMs like phenobarbital, an allosteric modulator and agonist of GABA_A_Rs [21]. A key mechanism underlying this shift is the developmental change in expression profiles of the membrane-localized chloride cotransporters, KCC2 and NKCC1. NKCC1 accumulates Cl^-^ intracellularly by pumping Cl^-^ into the cytosol, whereas KCC2 extrudes Cl^-^ from the cell in order to maintain a low [Cl^−^]_i_. In the mature brain, KCC2 is the dominant cotransporter, thereby permitting GABA_A_R action to be hyperpolarizing. In the neonatal and immature brain, KCC2 expression is insufficient to develop this Cl^-^ gradient, and GABA_A_R is found to be depolarizing [16]. This results in the neonatal seizure associated pharmacoresistance to ASMs such as phenobarbital [22].

Transient HIE seizures remit but carry a risk of long-term epilepsy, psychiatric comorbidities, and other neurological deficits. Curbing neonatal seizures effectively may help deter the development of long-term neurological diseases such as TLE. The mechanisms responsible for epileptogenesis following acute seizures are unresolved. Further, the time course of epileptogenesis appears to be both insult and subject-specific. Approximately 40% of TLE patients have refractory seizures, however the role of sex as a biological variable in the evolution of refractoriness remains unknown. Studies demonstrating the influence of sex on the time course of epileptogenesis after brain injury are lacking; future experimental paradigms powered by sex will help elucidate these mechanisms. Thus, one of the focuses of this review will be to examine the possible mechanisms governing refractoriness in neonatal seizures and TLE. Pathways known to be sexually dimorphic and involved in seizure severity and susceptibilities are discussed. 

The examination of epilepsy subtypes has demonstrated compelling sex differences [23]. HIE related seizures and its associated outcomes are sexually dimorphic, with a higher susceptibility and incidence observed in males [24,25]. In TLE, the generalization and lateralization of seizures is sexually dimorphic, with males experiencing tonic-clonic seizures at a greater rate than females [26]. The developmental expression of KCC2 and NKCC1 is also sexually dimorphic [27]; this suggests that sexually dimorphic modulation of chloride regulation may be one of the key mechanisms underlying differences in seizure susceptibility and refractoriness between sexes.

This review will seek to summarize literature concerning sex-dependent differences in the expression of neuronal Cl^−^ cotransporters, TrkB-BDNF, and other related pathways that directly influence neuronal [Cl]_i_. The proconvulsant and anticonvulsant roles of the gonadal sex hormones are also examined, particularly in sex-specific pathologies such as catamenial epilepsy. The mechanisms underlying the pharmacoresistance of HIE-associated seizures and TLE are elaborated in the context of the TrkB-BDNF pathway and its effect on KCC2 expression. Lastly, we briefly discuss epilepsy syndromes where the causes for the reported sex-differences are not clearly understood. The hope is that the care and management of those patients would benefit from an improved understanding of underlying sexually dimorphic mechanisms. 

## 2. KCC2-NKCC1

The electroneutral cation-chloride cotransporters (CCCs) of the *SLC12A* gene family utilize Na^+^-K^+^-ATPase-generated electrochemical gradients to transport chloride across cell membranes. In the CNS, NKCC1 cotransports Cl^-^, Na^+^, and K^+^ into the cell. KCC1, KCC2, KCC3, and KCC4 cotransport Cl^-^ and K^+^ out of the cell. The CCCs NKCC1, KCC1, KCC3, and KCC4 are ubiquitously expressed [28]. In contrast, the chloride exporter KCC2 is preferentially expressed in neurons and consists of multiple isoforms each with their own unique developmental profile [29]. During development, KCC2 expression undergoes a robust upregulation [30]. The mostly widely studied KCC2 isoforms are the full length transcripts KCC2a and KCC2b, which differ in their N-termini [31]. At birth, KCC2a and KCC2b expression was found to be equal in mouse brainstem neurons, and KCC2b expression was shown to increase postnatally [32]. Of all the chloride extruding CCCs, KCC2b remains the predominant isoform expressed in the mature brain and is the chief Cl^-^ extruder in mature neurons [33]. It is acknowledged that the physiological role of isoform KCC2a is not yet well understood [34]. Therefore, for the purpose of this review, KCC2 will denote KCC2b unless otherwise stated. 

The activities and functions of KCC2 are largely attributed to spatiotemporal control of KCC2 translation, as well as its post-translational phosphorylation and dephosphorylation [12,35]. The chloride extrusion function of KCC2 is mediated through phosphorylation of residue serine 940 (S940) [36]. Phosphorylation of the S940 residue of KCC2 increases its cell surface stability, thereby increasing the rate of ion transport [36]. Protein kinase C (PKC) has been demonstrated to phosphorylate S940, while increased Ca^2+^ levels from excessive NMDA receptor activity triggers protein phosphatase 1 (PP1) activation and subsequent dephosphorylation of pKCC2 S940 [37]. Dephosphorylation of residue S940 attenuates both surface expression and function of KCC2 [37]. As the dynamic control of [Cl^−^]_i_ is essential for cell volume regulation, the evolutionarily conserved WNK-SPAK/OSR1 (with no lysine kinase -SPS1-related proline/alanine-rich kinase and oxidative stress-responsive kinase 1) complex controls the phosphoregulation of the CCCs [38]. Cell shrinkage promotes activation of the WNK-SPAK/OSR1 complex that phosphorylates a group of threonine residues on NKCC1 promoting Cl^-^ influx while concurrently phosphorylating the threonine 1007 (T1007) residue on KCC2, thereby decreasing KCC2 membrane insertion, reducing chloride extrusion, and promoting cell swelling [39]. Ongoing research is focused on better understanding how the WNK-SPAK/OSR1 complex responds to osmotic challenges and changes to [Cl^−^]_i_ [38].

Immature neurons have low KCC2 expression levels and high [Cl^−^]_i_ that results in the depolarizing efflux of Cl^-^ after the activation of GABA_A_ receptors by GABA [40]. This excitatory GABAergic neurotransmission is critical for neuronal migration, maturation, and microcircuit integration in the immature brain [16]. The deleterious effects of KCC2 downregulation have been demonstrated using a *Kcc2^-/-^* mouse model; KCC2 knockout mice died shortly after birth due to motor deficits resulting in respiratory failure [41]. In a similar study using a KCC2 knockout mouse with ~5% greater levels of KCC2 expression, the mice exhibited spasticity, developed generalized seizures, and died in the third postnatal week [42]. During development, KCC2 expression increases as [Cl^-^]_i_ decreases, and GABAergic neurotransmission results in the hyperpolarizing influx of Cl^-^ into the neuron [30,40]. The development and maintenance of the neuronal chloride gradient is vital for the efficacy of GABA_A_-mediated inhibition [43]. This efficacious inhibition is critical for enabling diverse neural computations while preventing runaway excitatory activity such as seizures [44]. Disruption of the established balance between KCC2 and NKCC1 expression likely underlies the sexually dimorphic seizure susceptibilities observed in a diverse group of encephalopathies.

The functions of KCC2 extend beyond chloride extrusion activity; preclinical studies have demonstrated that KCC2 plays a critical role in the promotion of dendritic spine development through protein-protein interactions between the C-terminal domain of KCC2 and the cytoskeletal protein 4.1N [45]. Excitation in the CNS depends heavily on the shape and size of the dendritic spines, as the majority of glutamatergic synapses are formed on dendritic spines [46]. Aberrant dendritic spine morphology is associated with multiple neuropsychiatric disorders, including epilepsy and autism spectrum disorder [47]. Historically it has been difficult to determine the effect of dendritic spine abnormalities on the promotion of seizures as seizures themselves may cause damage to dendrites and dendritic spines, resulting in the development of hyperexcitable circuits and epileptogenesis [47]. Moreover, mechanisms of synaptic plasticity are affected by morphological changes in dendritic spines and are implicated in processes involving learning and memory, such as long-term potentiation [48]. It is evident that the role of KCC2 in epilepsy is multifaceted; a greater understanding of the structural and chemical functions of KCC2 will be critical in developing novel clinical approaches for epilepsy patients. 

**Figure 1:**
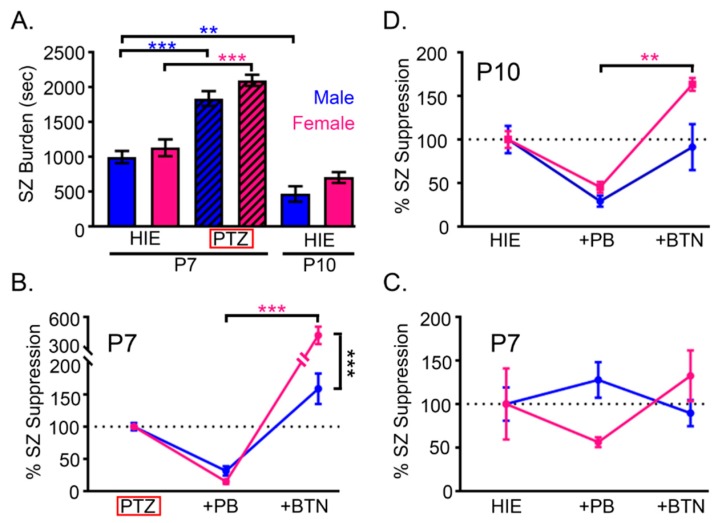
Sex-dependent developmental changes in neonatal seizure susceptibility and responses to antiseizure medications (ASMs) summarized from two neonatal seizure models [52,53]. (counter-clockwise) (**A**) During development males had a higher neonatal seizure susceptibility at P7 than P10 (P7 HIE Males vs. P10 HIE Males: One-way ANOVA; F_5,59_ = 19.44, *p* = 0.0044). In contrast, females did not have significant differences in age-dependent seizure susceptibility from P7 to P10. Males and females, both had significantly higher P7 seizure burdens when neonatal seizures were induced with chemoconvulsants (PTZ) when compared to unilateral carotid ligation (HIE)-induced seizures (P7 HIE Males vs. P7 PTZ Males: One-way ANOVA; F_5,59_ = 19.44, *p* = 0.0004; P7 Females vs. P7 PTZ Females: F_5,59_ = 19.44, *p* < 0.0001). (**B**) PTZ induced neonatal seizures at P7 were responsive to phenobarbital (PB) at 1 h after the initial PTZ injection. Bumetanide (BTN) administration at 2h resulted in significant seizure aggravation in females (P7 PB Females vs. P7 BTN Females: Two-way ANOVA; F_2,22_ = 14.6, *p* < 0.001). Aggravation of seizures by bumetanide was dependent upon sex as females had significantly greater seizure aggravation than males (P7 BTN Females vs. P7 BTN Males: Two-way ANOVA; F_2,22_ = 43.97, *p* < 0.001). (**C**) HIE induced seizures were non-responsive to PB in contrast to the PTZ induced seizures at P7. Neither PB nor BTN administration significantly suppressed HIE seizures in either sex. (**D**) HIE induced seizures at P10 are responsive to PB, with BTN administration significantly aggravating seizures in females (Two-way ANOVA; F_2,18_ = 2.334, *p* = 0.007). ** signifies *p* < 0.01; *** signifies *p* < 0.001.

As previously discussed, the developmental profile of KCC2 is sexually dimorphic. With regards to NKCC1, some evidence for developmental sex differences exists [27]. However, it is not clear which NKCC1 isoform is involved because there is a lack of studies that utilize isoform-specific probes [49]. Clinical trials that have used bumetanide, an FDA-approved loop diuretic and NKCC1/2 antagonist, for off-label treatment of neurological disorders have not reported sexually dimorphic outcomes as yet (for further details see review [49]). However, in preclinical studies, two different mouse models of neonatal seizures have observed seizure aggravation in females after BTN administration, and have been summarized here (Figure 1). The expression of KCC2 and NKCC1 is sexually dimorphic in the hippocampus and entorhinal cortex of neonatal rats. KCC2 remained consistently higher in females compared to males especially in the second post-natal week, in contrast NKCC1 peaks were detected only in males during the same period [27]. Although the expression of KCC2 mRNA increases throughout development in both males and females, it remains consistently higher in females [50]. Interesting new research also shows KCC2 downregulation in models of poststroke spasticity [51] however there are no insights into sexual dimorphism in the model.

Systemically, WNK1-mediated modulation of potassium channel function in mice is sexually dimorphic, with females exhibiting a lower blood pressure and higher plasma K^+^ concentration [54]. WNK-SPAK/OSR1 inhibition decreased epithelial NaCl reabsorption and K^+^ secretion to lower blood pressure while maintaining serum K^+^. In neurons, WNK-SPAK/OSR1 inhibition could facilitate Cl^-^ extrusion and promote GABAergic inhibition via the K^+^ driven CCCs. Estradiol administration to newborn rat pups increased the expression level of SPAK and OSR1 while enhancing the phosphorylation of NKCC1 [55]. These findings suggest that the WNK1-SPAK pathway, and ultimately the phoshoregulation of NKCC1 and KCC2, could be efficacious pharmacological targets to promote inhibition in epilepsy. WNK1-mediated control of the Ca^2+^-activated K^+^ channel function [56] suggests that the targeting of this pathway for inhibition would likely be more effective in females. During ischemia and repeated seizures, the extracellular concentration K^+^ can increase to high levels [57]. This pathological state induces astrocyte swelling via astrocytic NKCC1 activation. [58]. Blocking NKCC1 with BTN during high levels of extracellular K^+^ could hamper the ability for astrocytes to maintain low [K^+^]_o_ and osmotic homeostasis. Further, if the WNK1-SPAK pathway activation is sexually dimorphic during seizures, it could be one possible explanation for the BTN-mediated seizure aggravation detected in females mouse pups (Figure 1). Additional work investigating the role of sexual dimorphism in the phosphoregulation of NKCC1 and KCC2 during seizures is needed.

## 3. BDNF-TrkB Pathway Activation and Seizure Susceptibility

Neurotrophins are growth factors specific to neurons that regulate neuronal development and function. The neurotrophin family consists of nerve growth factor (NGF), brain-derived neurotrophic factor (BDNF), neurotrophin-3 (NT-3), and neurotrophin-4 (NT-4) [59]. Of these neurotrophins, BDNF is the most widely expressed throughout the CNS [60]. At the pre- and postsynaptic terminal of CA1 pyramidal neurons, BDNF acts as an autocrine signaling mechanism for the formation and maintenance of structural plasticity [61,62]. Cell-type specificity of BDNF-TrkB activity is demonstrated in a hippocampal feed-forward inhibition circuit where hippocampal mossy fibers synapse onto excitatory CA3 pyramidal neurons and inhibitory stratum lucidum interneurons (SLIN). After high frequency stimulation of mossy fibers, BDNF from mossy fiber presynaptic terminals mediates long-term depression (LTD) via retrograde endocannabinoid signaling between the SLIN and mossy fiber synapse. This LTD results in reduced excitatory drive onto SLIN, thereby reducing feed-forward inhibition onto CA3 pyramidal neurons and increasing the excitatory output of this circuit [63]. BDNF is also found in astrocytes and microglia, the latter being a cell-type that primarily performs a protective and defensive role within the CNS [64]. Signaling cascades that communicate the presence of potentially harmful conditions for neurons activate microglia by increasing the expression of purinergic receptor P2X4R. These receptors are activated by ATP, and promote BDNF release from microglia [65,66]. Studies in a rat model have demonstrated that exposure to 1L-1β, an endogenous cytokine protein, caused sex and region-specific differences on BDNF mRNA expression, with males experiencing significant attenuation in BDNF mRNA expression in the amygdala, hippocampus, and hypothalamus following 1L–1β exposure. BDNF mRNA expression in these regions remained unaffected in females; in contrast, a significant increase in serum BDNF levels was observed [67]. Therefore, BDNF has a broad range of functions some of which can be attributed to its activation of the TrkB pathway. 

BDNF binding to the TrkB receptor is responsible for increasing seizure susceptibility by the pathophysiological downregulation of KCC2 [13,51,68]. K252a, a TrkB receptor antagonist, produces a significant increase in KCC2 mRNA as well as protein levels in organotypic hippocampal slice cultures [68]. Upon TrkB dimerization and autophosphorylation, the phosphorylated residues activate the mitogen-activated protein kinase (MAPK) pathway [69]. Preclinical pharmacological strategies that inhibit BDNF-TrkB, or secondary messenger cascades that are required for TrkB activation after BDNF binding, have shown promise in curbing seizure susceptibility [13,70,71]. This novel strategy that helps maintain hyperpolarizing GABAergic inhibition could prove to be promising strategy to address refractory seizures both in neonates and in patients with TLE.

Some functions and mechanisms of BDNF vary in a sex-dependent manner as sex hormones and steroids are known to significantly modulate the activities of BDNF [59]. Comparisons of BDNF concentrations in male and female rat brains have shown higher levels of BDNF in the hippocampus, ventromedial hypothalamus, and cortex in female rats [72,73]. Sex and region-specific differences between *Bdnf* gene expression and estradiol concentration in the neonatal hippocampus of rats have been reported [74]. The same group found that male rats had an approximately 50% greater *Bdnf* gene expression in the dentate gyrus and CA1, but not CA3, than female rats. Moreover, treatment with estradiol resulted in differential effects on *Bdnf* gene expression amongst hippocampal subregions, but no differences on BDNF propeptide content [74]

Although there have been no studies demonstrating in vivo sexual dimorphism in BDNF-induced signaling to date, several recent studies have suggested that TrkB signaling is both region-specific and sex-dependent. It has been reported that male BDNF^+/-^ mice exhibited greater levels of TrkB tyrosine-705 (Y705) phosphorylation in both the frontal cortex and striatum than female BDNF^+/-^ mice at 10–16 weeks of age. Moreover, these sex differences were correlated with similar downstream changes in ERK2 phosphorylation in these regions [75]. In adult mice, the expression profile for TrkB phosphorylation in the CA1 stratum radiatum is dependent on the estrous cycle stage [76]. Specifically, these findings describe increased levels in axonal and glial pTrkB in proestrus females, and lower levels in estrus and diestrus females and males [76]. This suggests that an unknown sex-specific mechanism underlies this dimorphic TrkB signaling. The authors propose that these effects could be due to estradiol enhancing BDNF expression, resulting in a greater level of TrkB phosphorylation [77]. 

Estrogen is believed to interact with BDNF expression and signaling in several pathways. A seminal observation was that estrogen receptor mRNA is colocalized with *Bdnf*, *trk*, and *trkB* mRNA in cholinergic neurons during forebrain development [59]. Estrogen was later shown to regulate the expression of BDNF mRNA by demonstrating that a P0 gonadectomy resulted in reduced BDNF mRNA levels at P7 in male rat pups [78]. Treatment of gonadectomized animals with estradiol benzoate at P0 attenuated the effects of the gonadectomy, restoring BDNF mRNA levels [78]. In female mice long-term effects of ovariectomy have also shown increased methylation in the CpG islands of *BDNF* promoters IV and V in the hippocampus indicating a decrease in BNDF transcription [79]. However, the mechanism through which estrogen exerts this epigenetic control is yet unknown. A direct mechanism for estrogen’s induction of *BDNF* expression was described after the identification of an estrogen response element in the gene encoding BDNF [59], similar to the canonical estrogen response element traditionally found in estrogen-target genes, and confirmed that in vivo treatment with estrogen resulted in upregulated BDNF mRNA in the cerebral cortex of ovariectomized animals [59]. This research provides a foundation for our understanding of gonadal hormones and their effects on TrkB signaling. The effects of androgen concentrations have not been as thoroughly studied. Recently, it has been shown that testosterone upregulates expression of mRNA for both TrkB and BDNF in the spinal nucleus of the bulbocavernosus, and BDNF mRNA in the hippocampus CA1 area [80,81]. Although sexual dimorphism in TrkB signaling has been demonstrated throughout development, more research should be performed to elucidate the mechanisms that underpin these differences. For instance, the relationship of BDNF/TrkB signaling and the sex steroids is yet unknown. Preliminary studies have shown a relationship between cell proliferation due to changes in BDNF/TrkB signaling due to the presence of testosterone [82]. Moreover, gonadectomy resulted in attenuated expression of some BDNF transcripts in monkey and rat frontal cortices, and treatment with testosterone rescued these changes [83]. These results demonstrate that the mechanism at the root of these effects could be multifaceted, and a greater understanding of the role these hormones play could help in the advancement of treatments for pathologies correlated with aberrant levels of these proteins.

## 4. CCCs and Refractory Seizures

HIE composes the majority of neonatal seizures [6,84] that are notoriously resistant to first line antiseizure drugs such as phenobarbital [85,86]. The inefficacy of the first-line anticonvulsant PB has been proposed to depend on the reversal potential for Cl^−^ [1]. In neonatal seizure clinical trials, the NKCC1 inhibitor bumetanide (BTN) was used as an adjunct to phenobarbital [87]. This pharmacological treatment strategy failed to reverse phenobarbital refractoriness [87]. Out of the 14 babies, 10 were boys and the trial terminated prematurely due to a lack of seizure reduction and increased risk for ototoxicity [87]. BTN efficacy in preclinical models of chemoconvulsant-induced neonatal seizures provided the foundation for neonatal seizure clinical trials utilizing BTN [88]. Recent reports highlight the importance of understanding the effect of chemoconvulsants on CCCs in the immature brain [53]. Different induction paradigms for neonatal seizures have varying degrees of seizure burdens, refractoriness to phenobarbital, and sex-dependent differences (Figure 1). 

BDNF’s role in the promotion of seizure refractoriness is well investigated in the immature brain, and pharmacological strategies that target BDNF have had success [13]. Following ischemia and seizure-like activity, BDNF increases rapidly [89,90,91]. TrkB receptor activation by BDNF is associated with the downregulation of KCC2 [68]. ProBDNF, the precursor of BDNF, also downregulates KCC2 expression after binding to the ProBDNF receptor p75^NTR^ [92,93]. The increase of BDNF, proBDNF, and p75^NTR^ influence the downregulation of KCC2 that results in depolarizing GABAergic signaling, the loss of efficacious inhibition, and hyper-excitability [92]. In a preclinical model of HIE-associated neonatal seizures, refractory seizures are associated with younger ages of insult, in accordance with lower KCC2 expression levels [52]. ANA-12, a low-molecular weight TrkB receptor antagonist, when given as an adjunct to phenobarbital rescued refractoriness and KCC2 expression levels in a preclinical rodent model of HIE-induced neonatal seizures [13,70]. The acute, specific, and transient (i.e., with a single dose post-stroke) blocking of the TrkB receptor may be a novel and effective way of curbing the emergence of pharmaco-resistant seizures in the neonatal brain [70]. This transient block also rescued the acute and subacute downregulation of KCC2 which otherwise could be detrimental to brain maturation given the important role of KCC2 in dendritic spine formation, AMPA receptor traffic, and formation of functional GABA synapses [31,94,95]. Ischemia-related transient downregulation of KCC2 may also underlie the transient nature of HIE-related neonatal seizures in the first week of life. ANA-12 binds to the extracellular domain of TrkB, prevents BDNF-induced TrkB activation, and abolishes the biological effects of BDNF on TrkB-expressing cells but not those of NGF or NT-3 on TrkA- and TrkC-expressing cells. Therefore, sequestering BDNF from TrkB during the acute ischemic insult with this novel compound could add a new perspective for the development of therapeutic strategies in neonatal stroke management. Rescuing KCC2 downregulation could be a therapeutic path toward addressing pharmacoresistant refractory seizures. In addition to TrkB antagonists such as ANA-12, studies using a mouse model have shown that uncoupling PLCγ1 from TrkB following SE using a membrane-permeable peptide (pY816) inhibited TLE while maintaining the neuroprotective effects of TrkB signaling pathways [71]. These results are congruent with previous data suggesting that inhibition of TrkB prevented epilepsy caused by SE [96]. 

As previously mentioned, TLE can also result in refractory seizures that require surgical resective intervention to remove seizure focus. Resected tissue from patients with refractory TLE shows attenuated expression of KCC2 [11]. It is suggested that KCC2 hypofunction underlies the refractoriness observed in TLE. Double in situ hybridization studies of brain tissue from mesial temporal lobe epilepsy patients showed absence of KCC2 mRNA in 30% of CaMKIIα-positive subicular pyramidal cells [12]. Similarly, cortical tissues resected from patients with refractory seizures due to cortical dysplasia have also shown a reduction in membrane bound KCC2 [97]. In contrast, western blotting and immunocytochemistry reports of enhanced KCC2 expression in specific locations of the hippocampi resected from patients with human temporal lobe epilepsy [98] indicate KCC2 expression may be differentially up or downregulated both by location in hippocampus and on neuronal structure (i.e.; soma vs. dendritic spines). 

In male patients, the brain atrophy in TLE was associated with a greater number of generalized seizures compared to females and suggests that the male brain is more susceptible to seizure-related damage. 80% of patients with mesial TLE show interictal glucose hypometabolism [97]. Sexual dimorphism was also noted in the spread of hypometabolism, with males more often presenting frontal lobe hypometabolism and epileptiform activity ipsilateral to seizure onset, whereas females more frequently presented hypometabolism and epileptiform activity in the contralateral temporal lobe [97]. Magnetic resonance imaging in males with TLE were found to have greater ipsilateral and contralateral hemicranial volume loss than females with TLE [96]. Therefore, sex influences both the severity and degree of sclerosis in TLE.

## 5. CCCs and Febrile Seizures

One of the known initial insults that can result in TLE are febrile seizures (FS). FS are classically defined as seizures that occur in children with a temperature greater than ~38.4 °C (101.0°F) who do not have a history of metabolic disturbance, intracranial infection, or previous afebrile seizures [99]. The two general categories of FS are simple and complex, with further subdivisions of complex seizures such as febrile status epilepticus (FSE). Simple FS are relatively common events during childhood that have a generalized seizure onset, occur only once in 24 h, and last for less than 15 minutes [99]. In contrast, the prolonged seizure duration in FSE is a neurological life-threatening emergency with an increased risk for the development TLE later in life [100]. 

Ongoing clinical and preclinical research is focused on identifying the molecular mechanisms responsible for seizures during fever, the prolonged durations of seizures in complex FS, and the overall relationship of FS to epileptogenesis. The identification of mutations that are enriched in FS patient populations has demonstrated a role for KCC2 and GABA_A_ receptors in inherited cases of FS [101,102,103]. In rats, attenuation of KCC2 expression in hippocampal pyramidal neurons through in utero electroporation of shRNA reduced susceptibility to febrile seizures [104]. The developmental expression of KCC2 and GABA_A_ receptors are sexually dimorphic with males lagging behind females, therefore insight into sex-dependent differences of FS is warranted. In a clinical case-control study analyzing simple FS cases versus FSE cases, females were associated with an increased risk for FSE [105]. Preclinical FS research has demonstrated a higher susceptibility to PTZ and maximal electric shock induced seizures in female adult rats than males, when subjected to a model for complex FS during infancy [106]. The molecular mechanisms underlying epileptogenesis are a current field of focus in the field of epilepsy research. The incorporation of long-term electrographic monitoring [107] in preclinical studies examining hyperthermia-induced seizures will be of great benefit as how complex FS influences epileptogenesis is unknown [108].

## 6. Sexual Dimorphism and Seizure Susceptibility

Two major factors from which sex differences generally arise are sex chromosomes, steroid and peptide hormones. The gonadal steroids; androgens in males and estrogens in females, vary in concentrations between the sexes. Differences in the concentration of gonadal steroids can result in sexually dimorphic differences in structure and protein expression in the brain. Estrogens are known to modulate NMDARs. A study examining sex steroid hormone administration (estradiol or progesterone treatment in females, testosterone or dihydrotestosterone in males) observed that estradiol administration to ovariectomized wild type and BDNF^+/−^ female mice rescued expression of the NMDAR subunit GluN1 in the dorsal hippocampus of wild type mice but not in BDNF^+/-^ mice. This suggests that the effect of estradiol on NMDAR subunit expression is BDNF-dependent [109]. In both wild type and BDNF^+/-^ males, there was no observable effect of treatment with testosterone or dihydrotestosterone on GluN1 or GluN2A expression in the dorsal and ventral hippocampus. This indicates that sex-steroid modulation of BDNF mediated NMDAR subunit is sexually dimorphic. Mouse studies have demonstrated that androstenediol, a steroid hormone, activates GABA_A_ through allosteric modulation [110,111]. KCC2 mRNA has been shown to be upregulated by treatment with testosterone and dihydrotestosterone in the substantia nigra of both male and female rats [50]. Preclinical studies looking at downstream effects of perinatal inflammatory insults associated with early birth have shown broad impacts on neurodevelopment [112]. These insults showed sexual dimorphism with males displaying impaired performance in neurodevelopmental testing compared to females [112]. 

Infants exposed prenatally to intrauterine inflammation such as chorioamnionitis have a greater risk of developing complications such as epilepsy or cognitive deficits [113]. Early-life stress paradigms have been used extensively in studying epileptogenesis and seizures, as a growing amount of evidence suggests that stressors in early life are likely to accelerate epileptogenesis [114]. Prenatal stress has been shown to have sexually dimorphic effects on seizure susceptibility in a rat model; after 20 min of restraint stress on gestational day 18, offspring of stressed rats were more susceptible to kainic acid induced seizures, with males showing a greater susceptibility than females [115]. Clinical studies have shown that infants who experienced prenatal stress presented exacerbated febrile seizure duration and intensity [116], with males having a 1.75 times greater risk of experiencing a complex seizure. Generally, males have been found to be more susceptible to TLE-like seizures in chemoconvulsant exposure models. Rats treated with 10–12 mg/kg kainic acid showed a greater rate of full limbic convulsions in males [95]. The study examined the role of testosterone in the increased susceptibility to seizures and found a reciprocal relationship between chemoconvulsant-induced TL seizures and plasma testosterone [95]. It has also been shown that freeze-lesion induced cortical malformation at P1, followed by prolonged febrile seizures at P10 resulted in male rats developing temporal lobe epilepsy from P90–120, whereas female rats did not [117]. Male rats were also found to have elevated levels of the stress hormone corticosterone after the P1 lesion. A similar result was observed in female rats treated with testosterone [117]. 

It is hypothesized that neuroendocrine control exhibited by steroid hormones such as progestins and estrogens is a key factor in the etiology of catamenial epilepsy. Catamenial epilepsy affects women at a varying severity depending on the serum levels of the estrogens and progestins, which vary in concentration throughout the menstrual cycle [118]. Three types of catamenial seizures have been characterized: perimenstrual, periovulatory, and inadequate luteal [119]. The most common type of catamenial epilepsy seen in clinical settings is perimenstrual. Catamenial seizures, like HIE-associated seizures, are generally pharmacoresistant, though some women have shown state-dependent pharmacoresistance; improvements in seizure at certain points of the menstrual cycle have been observed [120]. Animals exposed to progesterone experience decreased convulsant-induced seizures [121]. In contrast, it has been demonstrated that estrogen functions as a proconvulsant, eliciting seizures or decreasing seizure thresholds in animal models [121]. Although it is evident that neurosteroids do have a sexually dimorphic impact on seizure susceptibility and KCC2 expression, the effect of neurosteroids specifically on chloride transport are not known. 

Greater understanding of the mechanisms underlying sex-dependent signaling in epilepsy is critical, as aside from the deleterious effects of the underlying pathology itself, epilepsy also has long-term comorbidities. Rolandic epilepsy, a form of epilepsy that tends to remit around age 14–18, also affects males at a greater rate than females. Children of both sexes with Rolandic epilepsy demonstrate lower scores on neuropsychological tests compared to healthy control subjects, and are more likely to develop attention-deficit hyperactivity disorder symptoms [122].

## 7. Conclusions

To summarize, seizure susceptibilities, epilepsy natural history, and phenotypes can be sex-dependent. This behooves clinical trials and preclinical studies to power study sample sizes to include equal numbers of both sexes when evaluating phenotypes, severity, mechanisms, and drug responses in epilepsy research. The National Institute of Health has now made it mandatory for studies not following this protocol to provide scientific explanations for exclusion criteria for either sex. Historically, preclinical studies in epilepsy research have systematically excluded females [123,124]. Emerging findings of mechanisms underlying sex-dependent differences in seizure susceptibilities and the evolution of epileptogenesis preclude direct comparisons to the findings of these past studies. Developmental susceptibilities tied to the expression profiles of Cl^-^ cotransporters and the pathways that modulate their membrane insertion and function show sexual dimorphism. Ongoing and future studies will help us better understand how sex may need to be incorporated as a biological variable into clinical practice and bed-side management.
